# Activity diversity is associated with the prevention of frailty in community-dwelling older adults: The Otassha Study

**DOI:** 10.3389/fpubh.2023.1113255

**Published:** 2023-03-23

**Authors:** Junta Takahashi, Hisashi Kawai, Manami Ejiri, Yoshinori Fujiwara, Hirohiko Hirano, Hiroyuki Sasai, Kazushige Ihara, Kaori Ishii, Koichiro Oka, Shuichi Obuchi

**Affiliations:** ^1^Research Team for Human Care, Tokyo Metropolitan Institute of Gerontology, Tokyo, Japan; ^2^Graduate School of Sport Sciences, Waseda University, Tokorozawa, Saitama, Japan; ^3^Department of Social Medicine, Hirosaki University Graduate School of Medicine, Aomori, Japan; ^4^Faculty of Sport Sciences, Waseda University, Tokorozawa, Saitama, Japan

**Keywords:** frailty, older adults, longitudinal study, activity, diversity

## Abstract

**Introduction:**

A prior study showed an association between diversity in daily activities (type, frequency, evenness) and frailty in older adults. However, the causality of this relationship is unclear. Therefore, this study aimed to clarify the relationship between activity diversity and frailty through a 2-year longitudinal study conducted among community-dwelling older adults.

**Methods:**

We evaluated data from the 2018 and 2020 waves of the Otassha Study. Frailty was assessed using the Cardiovascular Health Study criteria, with pre-frail and frail participants defined as frail and the other participants categorized into the robust group. We enrolled a total of 207 participants who were not frail at baseline. Activity type, frequency, and evenness scores were calculated using an Activity Diversity Questionnaire. The association between each activity diversity score and the incidence of frailty was evaluated using logistic regression modeling (each diversity score was entered the model after Z-transformation).

**Results:**

Of the 207 enrolled participants (median age, 73 years; age range, 65–91 years; 60.4% women), 64 (30.9%) had incident frailty during the follow-up period. A logistic regression analysis adjusting for sociodemographic and psychosomatic factors revealed odds ratios for activity type and evenness scores of 0.64 and 0.61, respectively (*P* < 0.05). These factors were significantly associated with the incidence of frailty.

**Discussion:**

Activity type and evenness (except frequency) within daily activities were predictors of frailty during 2 years of follow-up. Engagement in diverse activities appears to be more effective in preventing frailty than does engagement in a few activities.

## 1. Introduction

In developed countries, the burden of nursing care due to aging has become a major social problem, and extending healthy life expectancy is of urgent concern (1, 2). A decline in physical and mental functioning due to aging causes a decline in activities of daily living (ADL) and individuals' overall quality of life. Moreover, it burdens their families and the national medical care system as a whole (2).

Frailty is defined as an age-associated syndrome presenting as decreased reserve and resistance to stressors that cause vulnerability to adverse health outcomes (3). It is an important public health issue of increasing concern due to the global aging of the population (4). Previous studies have reported that frailty is associated with various health problems, including impairments in ADL, falls, institutionalization, hospitalization, and mortality risk (5). However, frailty has been reported to be reversible (4). Moreover, it might be possible to extend healthy life expectancy in at-risk individuals through appropriate interventions.

Factors have been reported to be associated with frailty, including sociodemographic, medical, physiological, and lifestyle factors (6, 7). In particular, lifestyle factors are modifiable and have attracted many researchers' attention. Previous studies have focused on daily activities, including physical, cultural, intellectual, and social activities. Daily activities have been reported to be associated with frailty in prior studies (8, 9). Although these studies examined associations between specific daily activity types and frailty, they did not evaluate daily activities comprehensively. As daily life consists of various activities, it is necessary to comprehensively consider these activities in terms of diversity and examine the association between the diversity of daily activities and frailty.

Several studies have examined associations between activity diversity and health outcomes. Menec (10) and Lee HY et al. (11) defined activity diversity as number of “types” of activities and reported that those who engaged in more types of activities had better cognitive function, less depression, and lower mortality rates. Verghese et al. (12) and Kesavayuth et al. (13) defined activity diversity as “frequency” of activities and reported that those who engaged in more frequency of activities were associated with better cognitive functioning and lower risk of dementia. In addition, Lee S et al. (14) defined activity diversity as “evenness” of engagement in activities over a week and reported that those who engaged in more evenness of activities had better mental health. The above studies have demonstrated that older adults who engage in diverse activities in their lives have better health conditions. However, few studies have examined their relationship with frailty.

In our previous study, we developed the Activity Diversity Questionnaire (ADQ), which is a questionnaire that assesses the implementation of 20 activities of daily living, including physical, intellectual, and social activities (15, 16). Then, we defined activity diversity as the type, frequency, and evenness of daily activities and conducted a cross-sectional study to examine the relationship between activity diversity and frailty in older adults. The results showed that activity diversity scores (including type, frequency, and evenness scores) were independently associated with frailty (17). However, this was a cross-sectional study, and the causal relationship between activity diversity and frailty was unclear.

The current study investigated the association between activity diversity scores and the incidence of frailty in community-dwelling older adults in a 2-year longitudinal cohort study aiming to clarify the causal factors in the association between activity diversity and frailty.

## 2. Materials and methods

### 2.1. Study design and participants

We conducted a 2-year longitudinal study using data from the Otassha Study cohorts. The Otassha Study cohorts is conducted among older adults aged 65 years or older living in nine areas of Itabashi Ward, Tokyo, except for those residing in nursing homes. Comprehensive health checkup surveys, consisting of a mail survey part and a venue survey part, are conducted in September and October every year (18, 19).

We enrolled individuals who participated in the 2018 survey. Individuals with frail or pre-frail at the time of the 2018 survey and those with missing data on outcomes or important covariates were excluded from the current study. In addition, individuals with cardiac disease or stroke in the past 6 months and with blood pressure above the reference value (systolic blood pressure ≥180 mmHg, diastolic blood pressure ≥100 mmHg) were also excluded as grip strength (the evaluation of which is required in the assessment of frailty) was not measured. All participants had been fully informed about the study (procedures, benefits, and drawbacks) and had provided their written informed consent prior to participation. This study was approved by the Ethics Committee of the Tokyo Metropolitan Institute of Gerontology (approval number: 2018–16) and was conducted in accordance with the principles of the Declaration of Helsinki.

### 2.2. Assessment of frailty

Frailty was assessed in the 2018 and 2020 surveys using the five items in the Japanese version of the Cardiovascular Health Study (J-CHS) criteria (20): weight loss, low activity, exhaustion, weakness, and slowness. Weight loss was defined as a loss of 2–3 kg or more in the past 6 months. Low activity was indicated by a negative response to both of the following items: “Do you engage in moderate levels of physical exercise or sports aimed at health?” “Do you engage in low levels of physical exercise aimed at health?” Exhaustion was indicated by a positive response to the following item: “(In the last 2 weeks) I have felt tired for no particular reason.” Weakness was indicated by a grip strength of < 26 kg for men and < 18 kg for women. Slowness was indicated by a usual gait speed of 1.0 m/s or less. Weight loss, low activity, and exhaustion were assessed in a mailed survey using a self-administered questionnaire, whereas grip strength and gait speed were measured at the survey site. Grip strength was measured twice using a hand dynamometer (Smedley-type hand dynamometer, Yagami, Nagoya, Japan). The strongest grip value of the two tests was considered for the analysis. Usual gait speed was obtained using a stopwatch to measure the time necessary to walk a 5-m walkway with 3-m acceleration and deceleration areas placed before and after the walkway. Walking speed was measured once. Those who met three or more of the above criteria were considered frail. Those who met one or two criteria were designated as pre-frail. Participants who were frail or pre-frail at baseline were excluded from the current study. Finally, those who developed pre-frailty or frailty during the 2-year follow-up period were allocated to the frail group and the rest to the robust group.

### 2.3. Assessment of activity diversity

Activity diversity was assessed using the Activity Diversity Questionnaire (ADQ) administered during the 2018 survey (10). The ADQ is a self-administered questionnaire that assesses 20 daily activities (see Supplementary Table 1). This questionnaire has been reported to have good reliability and validity within prior research (15). The 20 questionnaire items were preceded by the question: “How often did you engage in these activities in the last week?” The responses to the items were scored from 0 to 3, where 0 = “rarely,” 1 = “1–2 times a week,” 2 = “once every 2 days,” and 3 = “almost every day.” Activity diversity was defined as daily activities' type, frequency, and evenness (17). Scores for type, frequency, and evenness were calculated as follows. Type scores were calculated as the total number of activity types the individuals engaged in at least once a week; these scores could range from 0 to 20. Frequency scores were calculated by summing the frequency of engagement within the 20 activities (score range, 0–60). Evenness scores were calculated according to Shannon's entropy formula (21), as indicated by the following equation:


$$\begin{array}{l} \text{Evenness score}=-\left(\frac{1}{\log m}\right)\overset{m}{\underset{i=1}{\sum }}P_{i}\left(\log P_{i}\right) \end{array}$$

Where *m* represents the total number of activities (*m* = 20), *i* represents each activity, and *P*_*i*_ represents the proportion of each implemented activity. Evenness scores could range from 0 to 1, with higher scores indicating wide-ranging and even engagement in daily activities across the 20 evaluated items.

### 2.4. Covariates

Age, sex, the number of chronic diseases (stroke, heart disease, chronic obstructive pulmonary disease, chronic kidney disease, cancer, diabetes), subjective financial status (sufficient to live on, not sufficient to live on), and family structure (living with others, living alone) were examined as sociodemographic factors. In addition, physical and mental functioning were assessed using body mass index (BMI; < 18.5, 18.5–25.0, and >25.0 kg/m^2^), Mini-Mental State Examination (MMSE) (22), and the World Health Organization-Five Wellbeing Index (WHO-5) (23, 24). Subjective financial status and family structure were assessed through a mailed survey, whereas the other covariates were assessed at the survey site in 2018.

### 2.5. Statistical analysis

Differences in medical and demographic characteristics between the robust and incident frail groups were examined using *t*-tests and χ^2^ tests. Logistic regression analysis was performed to examine associations between activity diversity and the incidence of frailty, with the presence or absence of frailty as the dependent variable. To evaluate these associations, we implemented the following models: (i) a crude model, in which each of the three indicators of activity diversity (type, frequency, and evenness scores) were entered as independent variables; (ii) Model 1, in which sociodemographic factors were added as covariates to the crude model; and (iii) Model 2, in which physical and mental functioning factors were added to Model 1. The three indicators of activity diversity (type, frequency, and evenness score) were strongly correlated [type score vs. frequency score: *r* = 0.79 (*p* < 0.001), type score vs. evenness score: *r* = 0.98 (*p* < 0.001), and frequency score vs. evenness score: *r* = 0.85 (*p* < 0.001)], respectively. Due to multicollinearity issues, each indicator was not put into the model simultaneously. In the logistic regression analyses, the three indicators of activity diversity were standardized *via* Z-transformation to compare the odds ratios (ORs). Moreover, a power analysis was conducted using the G^*^Power software (25). Based on prior studies, the OR of the standardized activity diversity indicators with respect to the incidence of frailty or pre-frailty during study follow-up was presumed to be 1.5 (17). In contrast, the predicted incidence of frailty or pre-frailty was presumed to be 0.34 (26). Given an α of 0.05 and a targeted statistical power of 0.80, the minimum target sample size necessary within this study was *n* = 179.

We performed the following two supplementary analyses to examine the effects of those who were lost to follow-up and missing values. First, we compared medical and demographic characteristics at the baseline survey between the participants enrolled in the final analysis (*n* = 207) and those lost to follow-up (*n* = 119) using *t*-tests and χ-square tests. In addition, to ensure the robustness of the data in this study, missing value imputation was conducted supplementally using the multiple imputation method. The results with missing value imputation and the results from the complete case analysis were compared. Missing value imputation was performed on data from those who participated in both the 2018 and 2020 surveys (*n* = 456). Missing values were found in J-CHS criteria item [e.g., weight loss (*n* = 25), weakness (*n* = 47), slowness (*n* = 2) and low activity (*n* = 1)], subjective financial status (*n* = 34), family structure (*n* = 4) and MMSE (*n* = 1). Multiple imputation was performed using an iterative Markov chain Monte Carlo method (27). The number of imputed data sets was set to 100 (28), and logistic regression analysis was performed on the association between activity diversity and the occurrence of frailty, as in the main analysis. Since the missing value imputation was conducted as a supplementary analysis, only the results of the complete case analysis are shown in the main text.

The statistical significance level was set to a threshold of *P* < 0.05. All statistical analyses were conducted using IBM SPSS statistical software (version 28, IBM Corp. Armonk, NY, USA).

## 3. Results

**Figure 1:**
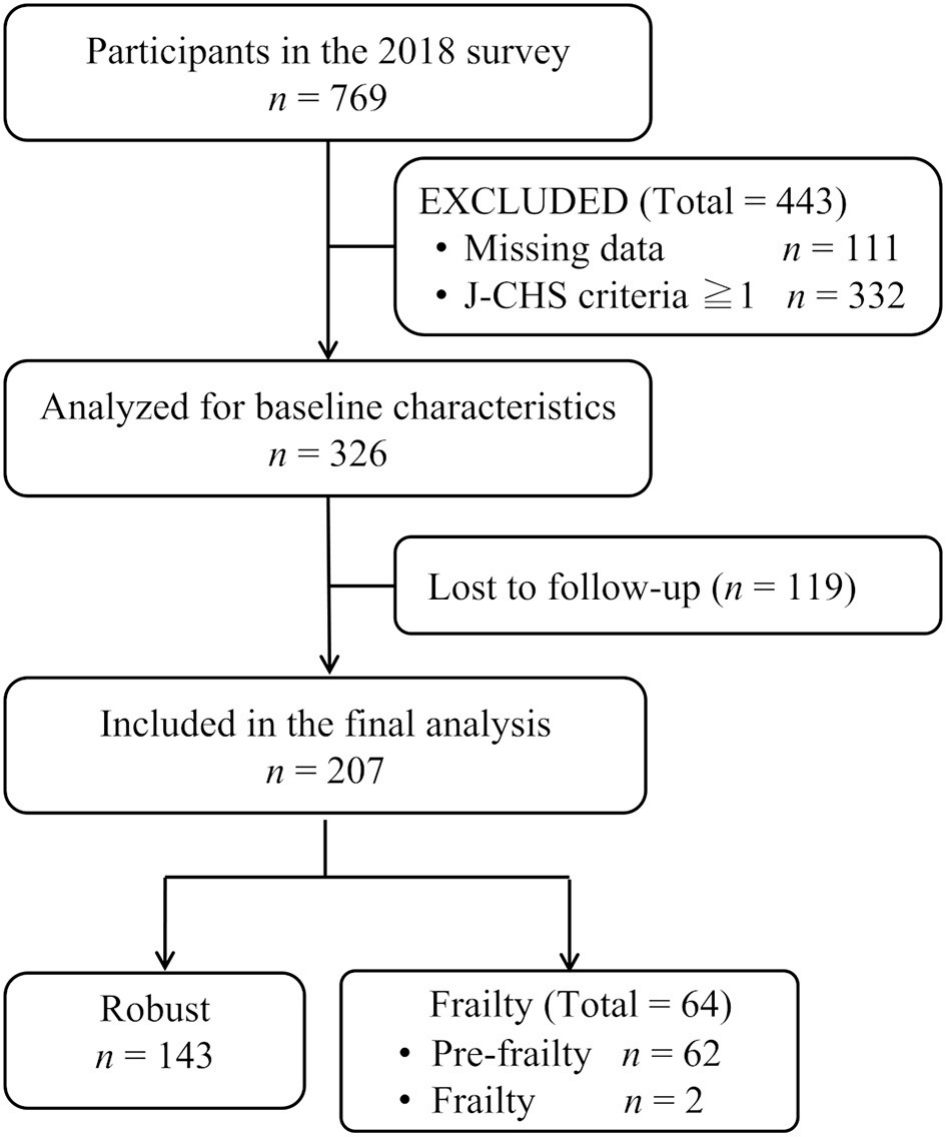
Flow chart of participant enrollment. J-CHS, Japanese version of the Cardiovascular Health Study.

The 2018 wave of the study included 769 participants, of whom 443 were excluded from the current secondary analysis due to the aforementioned exclusion criteria (missing data, 111; frail or pre-frail status, 332). One hundred nineteen participants were lost to follow-up as of the 2020 study wave. The final analysis included 207 participants [median age, 73 years; range, 65–89 years; 125 (60.4%) women]. Of the enrolled participants, there were 143 (69.1%) participants in the robust group as of the end of the study follow-up period and 64 (30.9%; pre-frail: *n* = 62, frail: *n* = 2) participants in the frail group (Figure 1).

Of the participants enrolled in the final analysis, 150 (72.5%) had an appropriate (normal) BMI (18.5–24.9 kg/m^2^). The mean MMSE score was 29.0 [standard deviation (SD) = 1.4]. The mean WHO-5 score was 18.5 (SD = 3.8). The evaluated activity type, frequency, and evenness scores were 11.2 (SD = 2.3), 24.4 (SD = 5.1), and 0.77 (SD = 0.07), respectively (Table 1). Type scores [robust vs. frail; 11.4 (SD = 2.2) vs. 10.6 (SD = 2.5), *P* = 0.015] and evenness scores [0.78 (SD = 0.07) vs. 0.75 (SD = 0.08), *P*=0.015] were lower in the frail group than in the robust group. There were no significant differences between the two groups with respect to the other evaluated items.

**Table 1:** Comparison of participants' medical and demographic characteristics in the 2018 survey.

**Variable**	**All** ***n*** = **207**		**Frailty status**				***P*-value**
			**Robust group**		**Frailty group**		
			***n*** = **143**		***n*** = **64**		
Age, years, median (range)	73	(65–89)	72	(65–89)	73	(65–84)	0.733
Women, *n* (%)	125	(60.4)	90	(62.9)	35	(54.7)	0.262
Number of chronic diseases, *n* (%)							0.222
0	110	(53.1)	81	(56.6)	29	(45.3)	
1	66	(31.9)	44	(30.8)	22	(34.4)	
2+	31	(15.0)	18	(12.6)	13	(20.3)	
Subjective financial status, *n* (%)							0.067
Sufficient to live on	189	(91.3)	134	(93.7)	55	(85.9)	
Not sufficient to live on	18	(8.7)	9	(6.3)	9	(14.1)	
Family structure, *n* (%)							0.111
Living with others	157	(75.8)	113	(79.0)	44	(68.8)	
Living alone	50	(24.2)	30	(21.0)	20	(31.3)	
BMI, kg/m^2^, *n* (%)							0.106
18.5–24.9	150	(72.5)	109	(76.2)	41	(64.1)	
< 18.5	14	(6.8)	10	(7.0)	4	(6.3)	
≥25.0	43	(20.8)	24	(16.8)	19	(29.7)	
MMSE, mean (SD)	29.0	(1.4)	29.1	(1.3)	28.9	(1.4)	0.352
WHO-5, mean (SD)	18.5	(3.8)	18.7	(3.8)	18.0	(4.0)	0.203
Type score, mean (SD)	11.2	(2.3)	11.4	(2.2)	10.6	(2.5)	0.015
Frequency score, mean (SD)	24.4	(5.1)	24.8	(5.1)	23.6	(5.3)	0.144
Evenness score, mean (SD)	0.77	(0.07)	0.78	(0.07)	0.75	(0.08)	0.015

BMI, body mass index; MMSE, Mini-Mental State Examination; SD, standard deviation; WHO-5, World Health Organization-Five Wellbeing Index.

A comparison of medical and demographic characteristics between the participants enrolled in the final analysis (*n* = 207) and those lost to follow-up (*n* = 119) are shown in Supplementary Table 2. Compared with the participants included in the final analysis, those who were lost to follow-up were significantly younger (final analysis vs. lost to follow-up, 72.8 (SD = 5.4) years vs. 71.2 (SD = 6.2) years, *P* = 0.013). No significant differences were observed with respect to the other evaluated items.

The results of logistic regression modeling showed that standardized type and evenness scores were significantly associated with the incidence of frailty in the crude model. This association was maintained after adjusting for all covariates specified within Models 1 and 2. The ORs for standardized type and evenness scores were ~0.6, indicating a similar degree of association with the incidence of frailty. Conversely, no significant associations were found for frequency scores within any model (crude model, Model 1, Model 2; Table 2).

**Table 2:** Results of logistic regression analyses with respect to the association between activity diversity and the incidence of frailty.

**Variable**	**Crude model**			**Model 1**			**Model 2**		
	**OR**	**(95% CI)**	***P*** **-value**	**OR**	**(95% CI)**	***P*** **-value**	**OR**	**(95% CI)**	***P*** **-value**
Standardized type scores	0.66	(0.47–0.93)	0.017	0.64	(0.45–0.91)	0.014	0.64	(0.44–0.92)	0.018
Standardized frequency scores	0.78	(0.56–1.09)	0.144	0.76	(0.54–1.07)	0.117	0.76	(0.53–1.09)	0.130
Standardized evenness scores	0.64	(0.44–0.92)	0.017	0.61	(0.41–0.90)	0.014	0.61	(0.40–0.91)	0.017

BMI, body mass index; CI, confidence interval; MMSE, Mini-Mental State Examination; OR, odds ratio; WHO-5, World Health Organization-Five WellBeing Index.

Crude model: Standardized type scores, standardized frequency scores, and standardized evenness scores were entered as independent variables.

Model 1: Adjusted for age, sex, the number of chronic diseases, perceived financial status, and family structure.

Model 2: Adjusted for the covariates in Model 1, plus BMI, MMSE, and WHO-5 scores.

When examining the association between activity diversity and the occurrence of frailty at 2 years in the data set with missing value imputation, a significant association was found between the type and evenness scores. On the other hand, the frequency score was not significantly associated with frailty (Supplementary Table 3). These results were similar to those of the main analysis using the complete case analysis method; therefore, only the results of the complete case analysis method are presented in the main text.

## 4. Discussion

The present study investigated associations between activity diversity and the incidence of frailty in community-dwelling older adults through a 2-year longitudinal study. Type and evenness scores were significantly associated with the incidence of frailty, even after adjusting for covariates such as sociodemographic variables and physical and mental functioning evaluations. While several prior studies have examined the association between frailty and specific daily activities (8, 9), few studies have focused on diversity in daily activities. To the best of our knowledge, this study is the first to show that activity diversity is a predictor of frailty, revealing the importance of maintaining and increasing the diversity of daily activities in older adults to prevent frailty.

The median age of the participants enrolled in this study was 73 years. Nevertheless, the percentage of participants with a normal BMI (18.5–24.9 kg/m^2^) was 75.8%, and the average scores for the MMSE and WHO-5 evaluations were 29.0 and 18.5, respectively. These findings indicate that the physical and mental functioning of these older participants was superior to that of participants evaluated in other cohort studies (23, 29–31). During the follow-up period, only two individuals became newly frail (vs. pre-frail) in this study (4.8 new cases per 1,000 person-years). This incidence of frailty was lower than that reported in a prior investigation (12.0 new cases per 1,000 person-years) (32). Thus, healthier older adults could have been recruited for this study due to the survey being conducted at a venue. Moreover, those with a deteriorated health condition (who were thus at a high risk of frailty) may have dropped out of the study during follow-up. However, it is important finding that even among these healthy older adults with a low risk of frailty, an independent association was found between activity diversity and the incidence of frailty (defined as frailty or pre-frailty). Although activity type and evenness scores at baseline were significantly lower in the frail group than in the robust group, the two groups were not significantly different with respect to the other evaluated characteristics. This suggests the importance of activity diversity in preventing frailty.

Logistic regression analysis demonstrated that both type and evenness scores were significantly associated with the incidence of frailty even after adjusting for all covariates, suggesting the importance of implementing more activities evenly. Seino et al. (33) investigated the association between the occurrence of functional disability and three healthy habits (moderate-to-vigorous-intensity physical activity, dietary variety, and social interaction). Their results showed that the risk of functional disability was reduced by 28% in those who had one type of habit, 49% in those who had two types of habits, and 62% in those who had all three types of habits compared to those who did nothing, suggesting that having a multidimensional lifestyle is important for the health of older adults. Ngandu et al. (34) examined the effect of a Multidimensional Lifestyle Improvement Intervention combining exercise, nutritional guidance, cognitive training, and social participation on cognitive function in the Finnish Geriatric Intervention Study to Prevent Cognitive Impairment and Disability (FINGER). They reported that the multidimensional approach to lifestyle was effective in reducing cognitive impairment. This, therefore, suggests that multidimensional lifestyle has positive impacts on health of older adults. Our findings showed that type and evenness scores were significantly associated with the occurrence of frailty. This suggests that those who engaged in more types of activities evenly were more likely to have a multidimensional lifestyle, and this may have been effective in preventing the occurrence of frailty. On the other hand, frequency scores showed no significant association with the incidence of frailty in the current study. Frequency scores were evaluated as the sum of the frequency of each activity; if the frequency of even one activity was high, the overall frequency score was high. Therefore, a high-frequency score does not necessarily indicate the implementation of multidimensional activities and may not be associated with the incidence of frailty (in contrast to the other two evaluated scores). In addition, the type score and evenness score were highly correlated (*r* = 0.98). This may be because the evenness score weights each activity according to the proportion of each implemented activity, but is also greatly influenced by the number of types of activities, which may have approximated the type score. The current operationalizations failed to empirically distinguish the two concepts, i.e., type and evenness. The two scores are theoretically distinct but methodologically not distinct. Therefore, it may be necessary to consider a new methodology for evenness indicator that is separated from the effects of number of types.

This study had some limitations. First, many participants were lost to follow-up (*n* = 119) or had missing data (*n* = 111). If the causes of dropout were related to the development of flailty, the bias due to lost to follow-up may have influenced the results of this study. The item with the highest amount of missing data was weight loss (*n* = 85, a sub-item of the J-CHS criteria), followed by subjective financial status (*n* = 58). The missing data was considerable, as information was obtained through a mailed survey. However, comparing the baseline characteristics of the participants included in the final analysis and those lost to follow-up showed only a significant difference in age. No significant differences in physical and mental functioning were observed. Furthermore, the analysis was also performed for the dataset with missing data imputation, and the results were similar to those of the main analysis. These results suggest a slight bias due to loss of follow-up and missing data. Moreover, we did not evaluate the amount of physical activity. A decreased amount of activity may affect the association between activity diversity and the incidence of frailty. Thus, it is necessary to simultaneously evaluate activity diversity and the amount of physical activity. Future studies enrolling more participants and intervention studies aimed at maintaining and improving activity diversity in older adults are warranted to confirm the findings of this investigation.

In conclusion, we found that activity type and evenness scores concerning daily activities were significantly associated with the incidence of frailty during a 2-year follow-up period, even after adjusting for a range of covariates. These results indicate the importance of diversity in daily activities (i.e., performing many types of daily activities evenly) to prevent frailty. Furthermore, our findings demonstrate the need for future efforts to maintain and improve the diversity of daily activities in older adults.

## Data availability statement

The datasets presented in this article are not readily available because of ethical and privacy restrictions. Requests to access the datasets should be directed to obuchipc@tmig.or.jp.

## Ethics statement

The studies involving human participants were reviewed and approved by Ethics Committee of the Tokyo Metropolitan Institute of Gerontology. The patients/participants provided their written informed consent to participate in this study.

## Author contributions

JT: formal analysis, investigation, and writing—original draft. HK: methodology, validation, investigation, resources, data curation, writing—review and editing, project administration, and funding acquisition. ME: project administration and investigation. YF, HH, HS, and KIh: investigation, resources, and funding acquisition. KIs: methodology. KO: supervision and methodology. SO: conceptualization, resources, supervision, and funding acquisition. All authors contributed to the article and approved the submitted version.
