# Supplementary material for: Activity diversity is associated with the prevention of frailty in community-dwelling older adults: The Otassha Study
Source: Front Public Health. 2023 Mar 23;11:1113255. doi: 10.3389/fpubh.2023.1113255 (PMC10078622; doi:10.3389/fpubh.2023.1113255)
Supplement: Supplementary file 1 [file Data_Sheet_1.pdf]

Supplementary Material

1 Supplementary Tables

Supplementary Table 1. Activity Diversity Questionnaire (ADQ)

	Almost every day	Every two days	1-2 days per week	Almost none
1. Inside chores<br>(e.g., cooking, washing, cleaning)	3	2	1	0
2. Outside chores<br>(e.g., purchasing daily necessities, gardening, house repairs)	3	2	1	0
3. Grooming<br>(e.g., dressing, make-up, shaving, hairdressing)	3	2	1	0
4. Screen time<br>(e.g., TV/ DVD/online program, surfing the internet)	3	2	1	0
5. Listening to the radio<br>(e.g., listening during work and/or travel)	3	2	1	0
6. Playing games<br>(e.g., boardgame, puzzle, computer/cellular phone game)	3	2	1	0
7. Gambling<br>(e.g., pachinko, horse racing, speedboat racing, bicycle racing, lottery)	3	2	1	0
8. Shopping<br>(e.g., for clothes, books, furniture, electrical appliances)	3	2	1	0
9. Direct contact with friends/relatives	3	2	1	0

**(e.g., conversations, shared meals, give advice)**				
**10. Indirect contact with friends/relatives**<br>**(e.g., through letters, talking on phone, e-mail, Internet calls)**	**3**	**2**	**1**	**0**
**11. Leisure activities with physically demanding components**<br>**(e.g., tennis, running, dance, walking, gymnastics)**	**3**	**2**	**1**	**0**
**13. Leisure activities without physically demanding components**<br>**(e.g., reading, music appreciation, playing instrument, flower arrangement, learning)**	**3**	**2**	**1**	**0**
**13. Regional activities/volunteering**<br>**(e.g., residents association, regional event, senior citizens' clubs)**	**3**	**2**	**1**	**0**
**14. Working**<br>**(e.g., part-time job, full-time work)**	**3**	**2**	**1**	**0**
**15. Childcare**<br>**(e.g., play together)**	**3**	**2**	**1**	**0**
**16. Elder care**<br>**(e.g., personal care, meal care, accompanying hospital visits)**	**3**	**2**	**1**	**0**
**17. Pet care**<br>**(e.g., walking pets, feeding, playing, grooming)**	**3**	**2**	**1**	**0**
**18. Hospital attendance**<br>**(e.g., dental clinic, acupuncture and moxibustion clinic, orthopedic clinic)**	**3**	**2**	**1**	**0**
**19. Driving cars/motorcycles**<br>**(excluding bicycles)**	**3**	**2**	**1**	**0**

20. Public transportation usage (e.g., train, bus, taxi)	3	2	1	0

Supplementary Table 2. Comparison of characteristics between participants in the final analysis and those lost to follow-up

Variable	Final analysis			Lost to follow-up		P-value
	n = 207			n = 119		
Age, years, mean (SD)	72.8	(5.4)		71.2	(6.2)	0.013
Women, n (%)	125	(60.4)		70	(58.8)	0.782
Number of chronic diseases, n (%)						0.128
0	110	(53.1)		72	(60.5)	
1	66	(31.9		38	(31.9)	
2+	31	(15.0)		9	(7.6)	
Subjective financial status, n (%)						0.676
Sufficient to live on	189	(91.3)		107	(89.9)	
Not sufficient to live on	18	(8.7)		12	(10.1)	
Family structure, n (%)						0.053
Living with others	157	(75.8)		101	(84.9)	
Living alone	50	(24.2)		18	(15.1)	
BMI, kg/m^2, n (%)						0.523

**18.5–24.9**	150	(72.5)		90	(75.6)	
**<18.5**	14	(6.8)		10	(8.4)	
**≥25.0**	43	(20.8)		19	(16.0)	
**MMSE, mean (SD)**	29.0	(1.4)		28.7	(1.5)	0.102
**WHO-5, mean (SD)**	18.5	(3.8)		18.0	(4.4)	0.252
**Type score, mean (SD)**	11.2	(2.3)		11.0	(2.2)	0.615
**Frequency score, mean (SD)**	24.4	(5.1)		24.1	(5.2)	0.648
**Evenness score, mean (SD)**	0.77	(0.07)		0.76	(0.07)	0.660

BMI: body mass index, MMSE: Mini-Mental State Examination, WHO-5: World Health Organization-Five Well-being Index, SD: standard deviation.

Supplemental Table 3. Results of logistic regression analyses with respect to the association between activity diversity and the incidence of frailty in the imputed data sets

**Variable**	**Crude model**				**Model 1**				**Model 2**		
	**OR**	**(95% CI)**	***P*-value**		**OR**	**(95% CI)**	***P*-value**		**OR**	**(95% CI)**	***P*-value**
**Standardized type scores**	0.63	(0.46-0.88)	0.01		0.60	(0.42-0.85)	0.004		0.60	(0.42-0.86)	0.01
**Standardized frequency scores**	0.75	(0.55-1.03)	0.08		0.72	(0.52-1.01)	0.05		0.72	(0.51-1.02)	0.06
**Standardized evenness scores**	0.61	(0.43-0.88)	0.01		0.57	(0.39-0.84)	0.004		0.57	(0.38-0.85)	0.01

BMI, body mass index; CI, confidence interval; MMSE, Mini-Mental State Examination; OR, odds ratio; WHO-5, World Health Organization-Five Well-Being Index

Crude model: Standardized type scores, standardized frequency scores, and standardized evenness scores were entered as independent variables.

Model 1: Adjusted for age, sex, the number of chronic diseases, perceived financial status, and family structure.

Model 2: Adjusted for the covariates in Model 1, plus BMI, MMSE, and WHO-5 scores.
